# Phospho-Protein Arrays as Effective Tools for Screening Possible Targets for Kinase Inhibitors and Their Use in Precision Pediatric Oncology

**DOI:** 10.3389/fonc.2019.00930

**Published:** 2019-09-20

**Authors:** Jakub Neradil, Michal Kyr, Kristyna Polaskova, Leos Kren, Petra Macigova, Jan Skoda, Jaroslav Sterba, Renata Veselska

**Affiliations:** ^1^Laboratory of Tumor Biology, Department of Experimental Biology, Faculty of Science, Masaryk University, Brno, Czechia; ^2^Department of Pediatric Oncology, Faculty of Medicine, University Hospital Brno, Masaryk University, Brno, Czechia; ^3^Department of Pathology, Faculty of Medicine, University Hospital Brno, Masaryk University, Brno, Czechia

**Keywords:** phospho-protein arrays, receptor tyrosin kinases, signal transduction, low-molecular-weight inhibitors, pediatric solid tumors, phosphorylation profiling

## Abstract

The specific targeting of signal transduction by low-molecular-weight inhibitors or monoclonal antibodies represents a very promising personalized treatment strategy in pediatric oncology. In this study, we present the successful and clinically relevant use of commercially available phospho-protein arrays for analyses of the phosphorylation profiles of a broad spectrum of receptor tyrosine kinases and their downstream signaling proteins in tumor tissue samples. Although these arrays were made for research purposes on human biological samples, they have already been used by several authors to profile various tumor types. Our study performed a systematic analysis of the advantages and pitfalls of the use of this method for personalized clinical medicine. In certain clinical cases and their series, we demonstrated the important aspects of data processing and evaluation, the use of phospho-protein arrays for single sample and serial sample analyses, and the validation of obtained results by immunohistochemistry, as well as the possibilities of this method for the hierarchical clustering of pediatric solid tumors. Our results clearly show that phospho-protein arrays are apparently useful for the clinical consideration of druggable molecular targets within a specific tumor. Thus, their potential validation for diagnostic purposes may substantially improve the personalized approach in the treatment of relapsed or refractory solid tumors.

## Introduction

Current curative treatment regimens for high-risk pediatric solid tumors consist of surgery, sometimes radiotherapy (to achieve adequate local control) and different intensive chemotherapeutic schedules, with a highly limited role of targeted agents thus far. Despite this multimodal approach, the rate of survival in patients suffering from refractory or relapsed solid tumors is still disappointing, and treatment is accompanied by many early and late side effects. This finding supports the need for more effective therapeutic approaches that are based on the principle of personalized medicine (1).

The personalized treatment of malignant diseases is defined as evidence-based, individualized medicine that delivers the right care to the right cancer patient at the right time (2). This personalized approach leads to measurable improvements in patient outcomes and thus to a rational distribution of health care costs (3). Therefore, such molecular individualized medicine has recently prevailed in traditional “one size fits all” medicine (2).

One very promising strategy involves the specific targeting of signal transduction by small molecule inhibitors or monoclonal antibodies; some of these medications have recently been tested in phase I and phase II clinical trials (4, 5). However, the basic step for this personalized approach includes the precise characterization of the individual tumor regarding the receptor tyrosine kinase (RTK) pattern—both the expression and phosphorylation correlating with activation—as well as of downstream signaling pathways. In the majority of published studies on this topic, total protein expression levels were usually considered, mostly on archival formalin-fixed paraffin-embedded (FFPE) tumor samples (6–8).

Nevertheless, a specific screening approach for activated, i.e., phosphorylated, RTKs and/or downstream signaling molecules should provide more accurate data concerning the dependency of tumor cells on a particular pathway and may provide a better guide for treatment choice (4, 9). In this article, we report the experimental use of commercially available phospho-protein arrays designed for the rapid screening of phosphorylated RTKs and other signaling molecules in several types of pediatric solid tumors. Although these arrays were made for research purposes on human biological samples, they have already been used for the characterization of certain tumors in adults (10, 11) and sporadically for the characterization of pediatric tumors (12–14). Nevertheless, no systematic analysis of the advantages and pitfalls of the possible use of this method for personalized clinical medicine is available. Thus, we hope that our results on the experimental use of this method may help validate its potential for clinical practice.

## Materials and Methods

### Tumor Samples

Tumor samples obtained from patients suffering from various types of relapsed or refractory pediatric solid tumors were included in this study. Written informed consent on the use of these samples and corresponding clinical data for research purposes were obtained from each patient or from the patient's parents/guardians. The Research Ethics Committee of the School of Medicine, Masaryk University (Brno, Czech Republic) approved the study protocol (certificate No. 29/2015). A description of the individual patients included in this study is given in Table 1. After surgery, the excised tumor tissue was examined macroscopically by pathologist and cut into two parts: one of them was designated for further microscopic examinations, and the second one was immediately frozen in liquid nitrogen. These frozen tumor samples were then processed for analyses using phospho-protein arrays. For immunohistochemistry (IHC) analyses, FFPE tumor samples were retrieved from files of the Department of Pathology, University Hospital Brno, Czech Republic.

**Table 1:** Overview of patients and their samples included in this study.

**Sample No**.	**Sample type**	**Patient age range (months)**	**Tumor histology**
1	Primary tumor	85–90	Malignant perivascular epitheliod cell tumor (PEComa)
2a	Primary tumor	185–190	Anaplastic ependymoma
2b	Relapsed primary tumor	215–220	
3	Relapsed primary tumor	35–40	Anaplastic ependymoma
4	Primary tumor	0–5	Infantile myofibromatosis
5	Primary tumor	20–25	Fibrodysplasia ossificans proggressiva
6	Lung metastasis	200–2005	Osteosarcoma
7	Primary tumor	95–100	Osteosarcoma
8a	Primary tumor	35–40	Alveolar rhabdomyosarcoma
8b	Relapsed primary tumor	45–50	
8c	Lymph node metastasis	45–50	
8d	Lymph node metastasis	80–85	
9	Primary tumor	20–25	Neuroblastoma
10	Primary tumor	30–35	Neuroblastoma
11	Orbital metastasis	15–20	Neuroblastoma
12	Hip metastasis	60–65	Neuroblastoma
13	Mediastinal metastasis	125–130	Neuroblastoma
14	Relapsed primary tumor	185–190	Pilocytic astrocytoma
15	Relapsed primary tumor	60–65	Pilocytic astrocytoma
16	Primary tumor	10–15	Pilomyxoid astrocytoma
17	Primary tumor	180–185	Glioblastoma
18	Relapsed primary tumor	140–145	Glioblastoma
19	Spinal cord metastasis	240–245	Medulloblastoma

### Phospho-Protein Array Analysis

The relative phosphorylation levels of the selected target molecules involved in signal transduction pathways in human cells were analyzed using two types of commercially available phospho-protein arrays (R&D Systems, Minneapolis, MN, USA). The Proteome Profiler™ Human Phospho-RTK Array Kit (R&D Systems, Cat. No. ARY001B) was designed for the parallel detection of the activities of 49 RTKs (Supplementary Table 1), and the Proteome Profiler™ Human Phospho-MAPK Array Kit (R&D Systems, Cat. No. ARY002B) was designed for the parallel detection of 26 downstream signaling molecules, including 9 MAPKs (Supplementary Table 2). Deeply frozen tumor tissue samples were cut with a scalpel in 400 μl of appropriate lysis buffer on ice. Lysis Buffer 17 and Lysis Buffer 6 (both R&D Systems) were used for Phospho-RTK and Phospho-MAPK array kit, respectively. After complete homogenization, the whole suspension was centrifuged for 5 min at 14,000 g. The supernatants were used as whole-tissue lysates and then processed according to the manufacturer's protocol. The levels of phosphorylation were quantified using ImageJ software (15). The detailed procedure of data acquisition and processing is described below in the relevant part of the Results.

### Immunohistochemical Analysis

Representative sections from relevant FFPE tumor samples were analyzed by IHC to determine the total and phosphorylated levels of the protein of interest. All of the antibodies used in this protocol are described in Table 2. The 4-μm-thick sections from FFPE blocks were deparaffinized with pure xylene for 3 × 5 min, washed in 96% alcohol for 3 × 5 min and then rinsed with distilled water. In the next step, endogenous peroxidase was inactivated by 3% H_2_O_2_ in methanol for 10 min, and the samples were washed in distilled water. Antigen retrieval was then performed by incubation in citrate buffer (pH 6.0) at 98°C for 20 min followed by cooling for 20 min and rinsing with PBS for 3 × 5 min. The incubation with primary antibodies was performed in a wet chamber at room temperature for 1 h, and samples were then rinsed with PBS for 3 × 5 min. The EnVision+System streptavidin-biotin peroxidase detection system (Dako, Glostrup, Denmark) was used according to the manufacturer's protocol in the wet chamber at room temperature for 45 min followed by washing in PBS and visualization using 3,3'diaminobenzidine as a substrate (Sigma-Aldrich, St. Louis, MO, USA). Nuclei were counterstained with Gill's hematoxylin for 1 min followed by bluing in water for 2–3 min for optimal results. Finally, the samples were dehydrated in a series of upconcentrated ethanol baths, cleared in xylene and mounted onto Entelan™ slides (Entelan Microscopy, Karlsruhe, Germany). Positive and negative controls were evaluated in each IHC run. Positive controls for each protein are listed in Table 2. Negative controls consisted of slides run without the primary antibodies. An Olympus BX45 microscope (Olympus Optical, Tokyo, Japan) equipped with an Olympus DP50 digital camera was used for the evaluation of IHC staining and to capture the micrographs. Olympus Viewfinder Lite™ software was used to process the images.

**Table 2:** Overview of antibodies used in this study.

**Antigen**	**Type/host**	**Cat. No. (all Abcam)**	**Dilution**	**Positive control**
EGFR (total)	Monoclonal/Rb	ab52894	1:200	Endometrial carcinoma
EGFR (anti-pTyr^1092^)	Monoclonal/Rb	ab40815	1:400	Papillary carcinoma of thyroid glad
PDGFRβ (total)	Monoclonal/Rb	ab32570	1:100	Breast and spleen
PDGFRβ (anti-pTyr^751^)	Polyclonal/Rb	ab51046	1:100	Brain
InsRβ (total)	Polyclonal/Rb	ab5500	1:200	Breast carcinoma
InsRβ (anti-pTyr^1185^)	Polyclonal/Rb	ab203278	1:150	Lung
Akt1/2/3 (total)	Monoclonal/Rb	ab32505	1:100	Prostate carcinoma
Akt1/2/3 (anti-pSer^473^)	Monoclonal/Rb	ab81283	1:50	Cervical carcinoma
ERK1/2 (total)	Monoclonal/Mo	ab54230	1:100	Stomach
ERK1/2 (anti-pThr^202^/pTyr^204^//pThr^185^/pTyr^187^)	Monoclonal/Mo	ab50011	1:100	Brain

### Statistical Analysis

Hierarchical clustering was used for multidimensional kinase phosphorylation data analysis to identify possible characteristic patterns of the kinase phosphorylation status for different diagnostic groups. Data preprocessing was performed as follows. Digitalized density levels were logarithmically transformed, and normalization and scaling of the data matrix were performed to enable comparison among samples. After filtering significantly different kinases, supervised clustering was applied. Hierarchical clustering using a Ward method with correlation distance metrics was performed. Data are presented using heatmap plots. Analyses were performed using R 3.4.3 (16) with gplots (17).

## Results

Although the phospho-protein arrays employed in this study were designed and produced by the manufacturer for research purposes only, our experimental results showed that they may also be successfully used for the rapid screening of active signal transducers as potential therapeutic targets in tumor tissue samples obtained from individual patients. Furthermore, we also showed that these arrays are also very suitable for comparative analyses of respective phospho-protein profiles among and/or within different tumor types, as described below in detail. Nevertheless, the use of phospho-protein arrays for these purposes encompasses several important aspects that must be carefully considered in the view of the correct interpretation of obtained results.

### Data Acquisition and Processing

**Figure 1:**
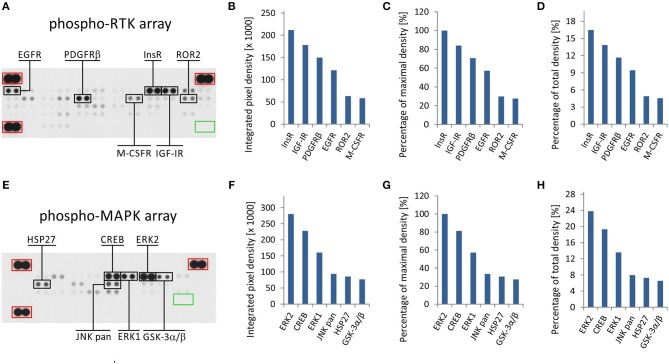
One sample analysis of phospho-RTK and phospho-MAPK array. The array images are shown for both arrays **(A,E)**, and the six most phosphorylated proteins are marked in black rectangles. Positive reference double spots are marked in red rectangles; area of negative control spots is marked in green rectangle. The most phosphorylated RTKs **(B–D)** and MAPKs or other downstream signaling molecules **(F–H)** are displayed according to intensity of phosphorylation. Absolute values of integrated pixel density **(B,F)** of different kinases are displayed also as a percentage of the maximal pixel density **(C,G)** or as a percentage of the total sum of pixel density **(D,H)** (y-axis).

The phospho-protein arrays used in our experiments are based on analysis of tissue samples on nitrocellulose membranes, where specific antibodies against selected kinases are spotted in duplicate. In addition to these antibodies, each membrane contains three positive reference double spots and one negative control containing PBS only, which is also spotted in duplicate (Figures 1A,E). Tissue lysates are applied to this membrane, and both phosphorylated and unphosphorylated proteins are bound to the respective antibodies at an equimolar ratio.

In the phospho-RTK array, the phosphorylated proteins are distinguished only by a pan-anti-phospho-tyrosine antibody conjugated with horseradish peroxidase. This allows us to detect all phosphorylated tyrosines that are predominantly located on the cytoplasmic part of the RTK molecule and thus reflect the overall activity of the receptor in question.

In the phospho-MAPK array, a mixture of biotinylated anti-phospho-kinase antibodies followed by streptavidin conjugated with horseradish peroxidase is used to distinguish between phosphorylated and unphosphorylated proteins. Each individual antibody in this antibody mixture is designed to detect a specific phosphorylation site (or sites) of each particular signaling protein included in the array. A table containing the overview of phosphorylation sites for all detected proteins is given in the manufacturer's manual.

Finally, the luminescence induced by the addition of a chemiluminescent substrate is captured on X-ray film in both arrays (Figures 1A,E). As described in our previous studies, the levels of phosphorylation were quantified using ImageJ software (15) and subsequently normalized to the positive control spots (18). Although the experimental use of a phospho-protein array is usually based on the comparison of acquired data with a reference cell line (18) or with untreated control cells (19), the employment of these arrays in clinical practice apparently requires a different approach.

We presumed that highly phosphorylated proteins, as detected by these arrays, are highly activated within the tumor tissue and thus represent the most suitable targets for treatment with available small-molecule inhibitors or monoclonal antibodies (20). To evaluate the results of one sample analysis, it is possible to use the absolute values of integrated density as obtained by the employment of image analysis software (Figures 1B,F). Alternatively, the data can be normalized to the maximal integrated density achieved in each individual array, i.e., the highest value indicates 100% phosphorylation (Figures 1C,G). The third method by which to process the obtained data is recommended by ImageJ software documentation (15): the integrated densities of each kinase are displayed as a percentage of the total sum of density (Figures 1D,H). The resulting phosphorylation profiles obtained by these three processing modes are identical to each other, and they show the same differences in the phosphorylation of kinases included in the respective array (Figures 1B–D,F–H).

From the clinical viewpoint, the results described above (Figure 1) are those obtained by the analysis of tumor tissue obtained from patient No. 1 (Table 1). This sample was obtained by surgical resection of the tumor mass from the supravesical space. According to the results from molecular biology analyses, including these phospho-profiles, the patient was treated with low-molecular-weight inhibitors (everolimus and sunitinib), and complete remission was achieved.

The results from RTK phospho-protein arrays showed that two members of the insulin receptor family (InsR and IGF-1R) displayed the highest phosphorylation. Slightly reduced positivity was also observed for PDGFRβ and EGFR (Figures 1A–D). The analysis of MAPKs revealed the high phosphorylation of ERK1 and ERK2 on activation loop residues Thr^202^/Tyr^204^ and Thr^185^/Tyr^187^, respectively. Among the other downstream signaling molecules, CREB was substantially phosphorylated on residue Ser^133^. Lower but still detectable levels of phosphorylation were found for JNK kinases on Thr^183^/Tyr^185^ and Thr^221^/Tyr^223^, for Hsp27 (also known as HSPB1) on Ser^78^/Ser^82^ residues, and for GSK-3 kinases on Ser^9^/Ser^21^ (Figures 1E–H).

### Verification of Druggable Targets in Tumor Tissue

The main advantage of the use of phospho-protein arrays for the identification of active signal transducers within tumor tissue is the promptness of such a screening method and the relatively high number of signaling molecules covered by these arrays. Nevertheless, as these arrays are still available for experimental purposes only and not for routine laboratory diagnostics, the results obtained from these arrays should be verified by other independent methods.

Thus, we employed a standard IHC method for the independent detection of active signal transducers as already identified by the phospho-protein array. As an example of this approach, the results from both types of phospho-protein arrays were compared with results from the IHC analysis of paired FFPE samples from the same biopsies in a group of 6 patients: patients No. 2–7 (Table 1) were included in this part of the study.

**Figure 2:**
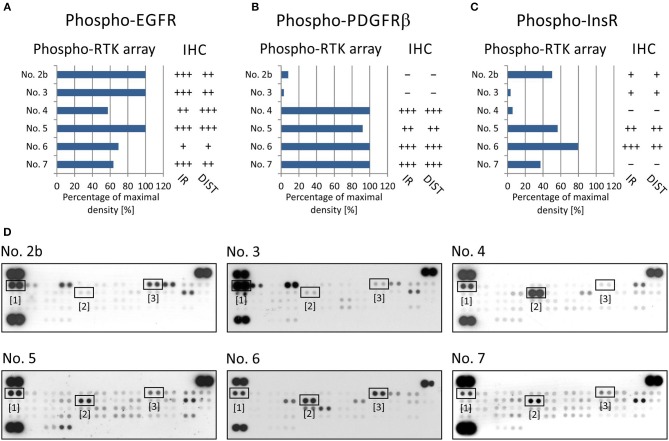
Comparison of immunohistochemical and phospho-protein array analysis in determination of phosphorylated EGFR **(A)**, PDGFRβ **(B)**, and InsR **(C)**. Evaluation of phospho-protein array: values are displayed as a percentage of the maximal pixel density of every single sample. Evaluation of IHC: intensity of immunoreactivity (IR); –, very weak; +, weak; ++, medium; +++, strong; distribution of immunostaining (DIST); –, non-detectable; +, focal; ++, regional; +++, diffuse. The images of phospho-RTK arrays **(D)** with marked phosphorylated EGFR (1), PDGFRβ (2), and InsR (3) proteins.

For RTKs, the levels of phospho-EGFR, phospho-PDGFRβ, and phospho-InsR were compared with the presence of these phosphorylated RTKs as identified using specific anti-phospho antibodies (Table 2) against these three selected target molecules (Figure 2).

To verify EGFR phosphorylation, a specific anti-phospho-EGFR antibody against phosphorylated Tyr^1092^, which is equivalent to Tyr^1068^ of mature EGFR, was used. All analyzed samples showed relatively high levels of phosphorylation as detected using both phospho-arrays, with ~60–100% of maximal density, and the immunoreactivity was predominantly medium to strong except in sample No. 6 (Figure 2A). The anti-phospho-PDGFRβ antibody against phosphorylated Tyr^751^ also showed a very good match with PDGFRβ phosphorylation as detected by the phospho-protein array in all six samples (Figures 2B,D): strong or medium immunoreactivity corresponded to the highest density from the phospho-protein arrays and vice versa; and very weak immunoreactivity was in accordance with very low phosphorylation—up to 10% of maximal density—in sample Nos. 2 and 3 (Figures 2B,D). The anti-InsR antibody against phosphorylated Tyr^1185^ in the beta chain of the InsR molecule showed strong or medium positivity in the same tumor tissues in which at least 50% of maximal density was detected by the phospho-array (Figures 2C,D). Nevertheless, for InsR activities up to 50% of maximal density, accordance with the IHC results was not obvious (Figures 2C,D).

**Figure 3:**
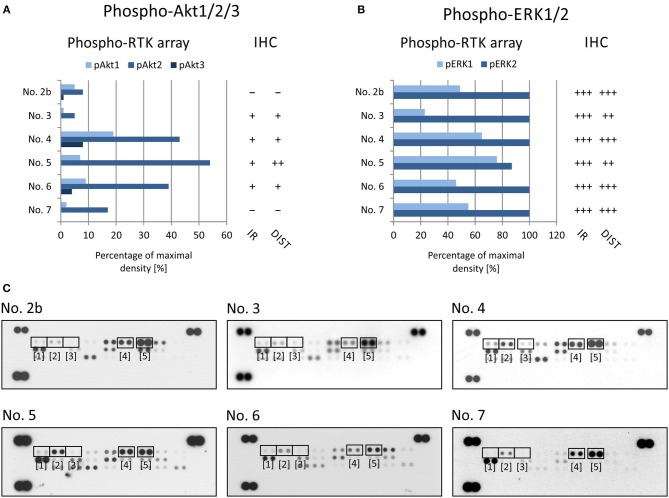
Comparison of immunohistochemical and phospho-protein array analysis in determination of phosphorylated ERK1/2 **(A)** and Akt1/2/3 **(B)**. Evaluation of phospho-protein array: values are displayed as a percentage of the maximal pixel density of every single sample. Evaluation of IHC: intensity of immunoreactivity (IR); –, very weak; +, weak; ++, medium; +++, strong; distribution of immunostaining (DIST); –, non-detectable; +, focal; ++, regional; +++, diffuse. The images of phospho-MAPK arrays **(C)** with marked phosphorylated AKT1 (1), AKT1 (2), AKT1 (3), ERK1 (4), and ERK2 (5) proteins.

To evaluate the two most prominent downstream signaling pathways, i.e., PI3K/AKT and RAS/RAF/MEK/ERK, antibodies designed to detect both phosphorylated forms of ERK kinase and all three phosphorylated forms of AKT kinase, respectively, were chosen. The phosphorylation of AKT kinases in FFPE tumor samples was evaluated using an anti-AKT1 antibody that detects phosphorylation at Ser^473^ within the C-terminus. Due to the high degree of similarity to the corresponding regions in the AKT2 and AKT3 molecules, this antibody may cross-react with these isoforms at Ser^474^ and Ser^472^, respectively. The phospho-protein array can detect the relative phosphorylation of all three AKT isoforms at the same phospho-sites as the antibody employed for IHC. Anyway, the phosphorylation of AKT2 reached only ~50% of the maximal density in sample Nos. 4, 5, and 6 (Figures 3A,C). The relative phosphorylation of AKT1 and AKT3 was close to the detection limit of this array in all tested samples, and the IHC method also showed very poor results for phosphorylated AKT1/2/3 molecules (Figures 3A,C). The anti-ERK1/2 antibody was designed against the epitopes with the same phospho-sites as those detected by the phospho-array. All FFPE samples showed strong immunoreactivity for ERK1/2, with diffuse or regional positivity, and these results were in accordance with the very high relative phosphorylation of ERK2 as detected by the phospho-protein array (Figures 3B,C).

Taken together, these results indicate that the IHC method using compatible anti-phospho antibodies can serve as a useful tool for final detection or rather confirmation of the phosphorylation of the possible therapeutic target previously identified in the tumor tissue by rapid screening using phospho-protein arrays.

### Example of Serial Sample Analysis During Clinical Progression of the Disease

As described above, the analysis of one individual tumor sample provides us with information concerning the phosphorylation profile of RTKs and/or downstream signaling molecules just at the time of tissue sample acquisition. A typical clinical reason for a one sample analysis is the rapid screening of suitable (and druggable) targets for personalized treatment during the phase of initial diagnostics. Nevertheless, especially in cases of refractory or relapsed tumors, we are also able to analyze and compare a series of tumor samples taken from the same patient at different phases of the disease.

**Figure 4:**
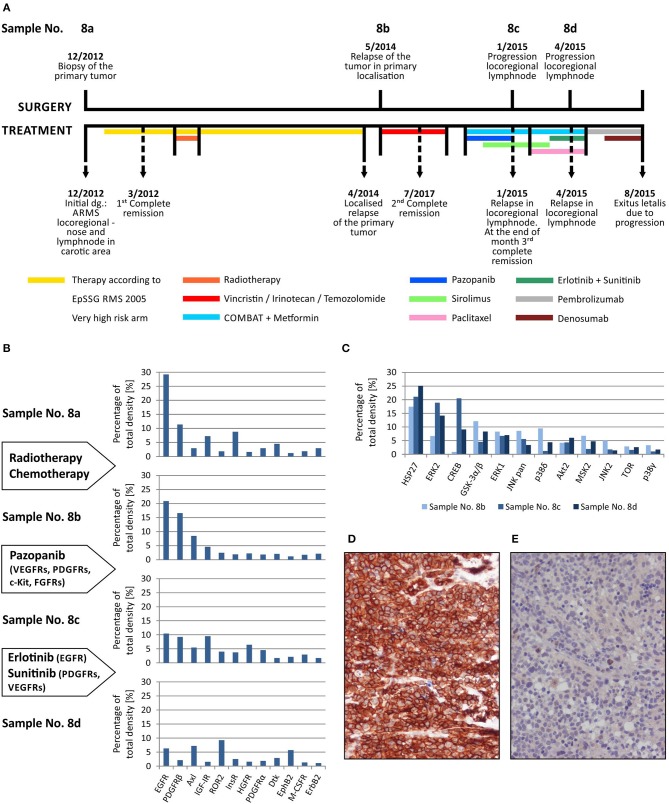
Sequential analysis of phospho-protein arrays during individualized therapy of alveolar rhabdomyosarcoma. Timeline of patient's surgical and medical treatment **(A)**. Changes in the phospho-RTKs profiles of samples obtained during therapy **(B)**. Changes in the phospho-MAPKs profiles of samples obtained during therapy **(C)**. Immunohistochemical detection of phospho-EGFR in sample No. 8b **(D)** and phospho-PDGFRβ in sample No. 8d **(E)**.

Here, we describe the employment of phospho-protein arrays in the profiling of cell signaling pathways in four serial tumor samples taken from a child suffering from PAX3/FKHR-positive alveolar rhabdomyosarcoma of the ala of nose. The child was diagnosed at the age of 19 months, staged as T1aN1M0 and classified into a very-high-risk group (IRS st. IIIa) according to the European Pediatric Soft Tissue Sarcoma Study Group (EpSSG). First, complete remission was achieved after 3 cycles of chemotherapy (EpSSG RMS2005 protocol) and adjuvant radiotherapy; however, the tumor relapsed after 16 months. Second, complete remission was achieved after 6 cycles of chemotherapy (vincristine, irinotecan, and temozolomide). The patient was treated with individualized metronomic chemotherapy, but metastatic relapse was diagnosed 3 months later. Despite intensive individualized therapy, the child died within 7 months. The complete overview of this case is given (Figure 4A).

Step-by-step, we analyzed using both phospho-protein arrays the samples taken from the primary tumor before the treatment using both phospho-protein arrays, from the relapsed primary tumor and from two metastatic lymph nodes at different times during the disease (Table 1, Figure 4A). Changes in the phospho-profiles of RTKs (Figure 4B) are described in the context of personalized therapy with small molecule inhibitors used in this patient; the phospho-profiles of downstream signaling pathways (Figure 4C), as well as examples of target validation by IHC (Figures 4D,E), are also given.

Based on the obtained data, specific small molecule inhibitors were incorporated into the treatment protocol. Although pazopanib showed partial effect in terms of RTK activity, the subsequent treatment with erlotinib and sunitinib markedly diminished the activities of the target RTKs in the tumor tissue (Figure 4B). Unfortunately, despite this very clear response at the molecular level, no encouraging effects of this targeted therapy were observed at the clinical level, probably because of the advanced stage of metastatic disease. Nevertheless, this case markedly illustrates the importance and usefulness of the rapid screening of the possible molecular targets for personalized therapy.

### Multidimensional Analysis of Kinase Profiles in Specific Tumor Types

**Figure 5:**
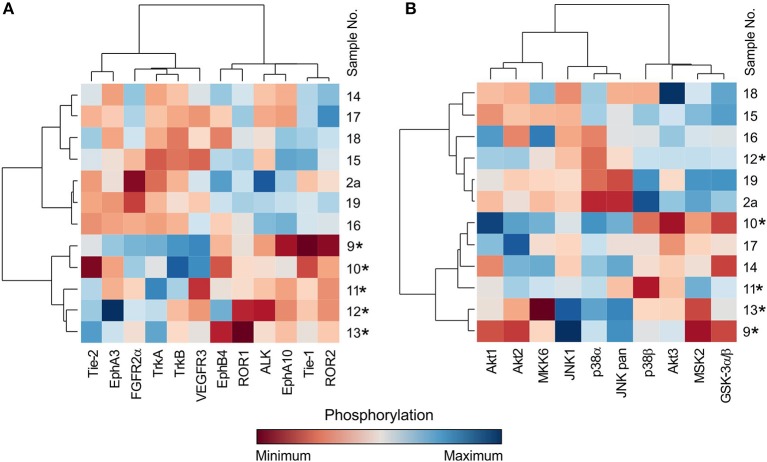
Cluster analysis of phospho-RTK **(A)** and phospho-MAPK **(B)** array data. Samples were obtained from patients with neuroblastoma (marked with star) and different types of brain tumors. Filter *p* < 0.05 significance.

In addition to the individual and serial sample analyses described above, information regarding the phosphorylation profiles as obtained by the phospho-protein arrays can also be used for the hierarchical clustering of selected tumor samples. Here, we demonstrate the performance of such a classical multidimensional analysis using supervised hierarchical clustering on a small cohort (*n* = 12) of 5 neuroblastoma and 7 central nervous system (CNS) tumor samples: 3 astrocytomas, 2 glioblastomas, 1 ependymoma, and 1 medulloblastoma. The patients' detailed information is given in Table 1. This analysis performed on the data from the RTK phospho-protein arrays showed two distinct clusters of strongly and weakly phosphorylated RTK kinases in neuroblastoma and CNS tumors (Figure 5A). In contrast, even supervised clustering based on the data from the MAPK phospho-protein arrays did not result in distinct clusters of diagnostic groups in the same cohort (Figure 5B).

## Discussion

An antibody array is one of the simplest methods for measuring the relative levels of expression or phosphorylation of several proteins in a single sample. In this study, we present the successful and clinically relevant use of the Human Phospho-RTK Array Kit and the Human Phospho-MAPK Array Kit (both by R&D Systems) for the analyses of the phosphorylation profiles of a broad spectrum of RTKs and their downstream signaling proteins.

According to the manufacturer's instructions, the analysis of raw data obtained from these arrays includes determining the average signal of the pair of duplicate spots and subtracting the background signal. However, the subsequent analysis of these data is neither unified nor standardized and thus depends on the researcher's choice.

The results of one sample analysis can be presented as a specific profile with differently phosphorylated proteins, in which high levels of the detected signal, i.e., high density of spots in the phospho-protein arrays, correspond to high phosphorylation. Consequently, these highly phosphorylated signaling molecules can be considered potential therapeutic targets for low-molecular-weight kinase inhibitors or monoclonal antibodies. Moreover, the stability of phosphorylation profiles of frozen samples throughout long-term storing was proven (Supplementary Figure 1). The utility of commercially available phospho-protein arrays has already been demonstrated in other studies on various types of human solid tumors in adults (10, 11, 21–23).

This experimental approach was also successfully used by our team in describing molecular targets and the subsequent effective treatment of several pediatric malignancies, such as Maffucci syndrome, which is characterized by multiple hemangiomas and enchondromas with a tendency to progress into malignancy (12), infantile myofibromatosis, in which PDGFR beta hyperphosphorylation is detected (13), or fibrodysplasia ossificans progressiva (14).

In addition to these individual cases, in this article, we summarize our experience with determining kinase phosphorylation profiles for single sample (Figures 1, 2) and serial sample (Figure 4) analyses. The promising clinical response of patient No. 1 to sunitinib administration and the changes in serial kinase profiles after treatment with targeted low-molecular weight inhibitors (Figure 4) are other positive examples of the rational use of this experimental approach as a rapid screening method for the identification of druggable targets, which is a key part of personalized therapy. Nevertheless, as this method is not certified for diagnostic purposes, it is of high importance to employ another validation method to confirm the data from phospho-protein arrays independently.

Our data from the IHC validation showed good consistency in the levels of the phosphorylated forms of all three selected RTKs, i.e., phospho-EGFR, phospho-PDGFRβ, and phospho-InsR, as determined independently by IHC and the phospho-protein array (Figure 2). Similarly, data on the downstream signal transducers ERK and AKT showed moderate accordance (Figure 3), although the pan-phospho-ERK and pan-phospho-AKT antibodies were used for IHC detection, whereas isoform-specific antibodies against ERK1/2 or AKT1/2/3 were spotted onto the MAPK phospho-protein array. The same strategy, i.e., the validation of phosphorylated signal transducers as suitable targets by IHC, was successfully used for personalized treatment with low-molecular-weight inhibitors in malignant mesothelioma (24).

The most interesting finding from our study is the example of a cluster analysis performed in a cohort of 12 patients suffering from neuroblastomas or CNS tumors. In general, for the graphical presentation of results on differences among individual samples and their clusters, a heat map is the best choice (Figure 5). For the heat map display, data normalization is required, and the values range from −3 to 3 (23, 25). The same approach was used, and the hierarchical clustering of data from the MAPK phospho-protein arrays showed no distinct clusters for these tumor types (Figure 5B), whereas the same clustering method revealed significantly different patterns of the selected 12 RTKs in neuroblastomas and in the group of CNS tumors (Figure 5A). These interesting data will be reanalyzed in our forthcoming study on a large cohort of patients with neurogenic tumors; however, these results suggest another useful approach to employ the phospho-protein arrays in personalized therapy.

As apparent from all results presented in the current study as well as those from previously published data obtained by the same type of phospho-protein arrays, the key step in the use of these arrays for the identification of druggable molecular targets is the manner of data processing and interpretation. Comparative analyses of phosphorylation profiles in various tumor tissue samples are typically used in these studies: the analysis of 20 glioma cell lines and 14 tissue samples of primary glioblastoma multiforme can be used as an example (26). The categorization of achieved data into several groups according to the signal intensity is also a frequently used approach (21, 22, 27). Some of these groups are distinguished by different levels of positive signals, and the last one is considered negative. The definition of a particular group can be described by specific categorization terms such as “strongly activated,” “moderately activated,” “activated to a lower extent,” and **“**poorly activated” (22) or by a grading system similar to the IHC evaluation (27). A simplified binary view is able to distinguish positive or negative signals only, and thus activated and non-activated proteins can be described by this approach (10, 11, 24). In the first of these studies, all intensity values of the probes and the local background of the probes were log_2_ transformed (to obtain a more symmetrical distribution) and subtracted. In the next step, the mean of all obtained values was calculated for the individual array, and only the probes with values higher than the mean plus standard deviation (SD) were considered significantly activated (10). In the second study, the cut-off level for activated proteins was calculated as triple that of the highest negative control (24). In the last study, the cut-off level was not described (11).

In conclusion, our study showed the usefulness of phospho-protein arrays for the personalized treatment of patients suffering from relapsed/refractory solid tumors. From the clinical point of view, these arrays are especially suitable for the rapid screening of targets for treatment with low-molecular-weight inhibitors or monoclonal antibodies, although they can also be used for deep analyses of the differences in phosphorylation profiles among selected tumor types. These phospho-protein arrays are available for research use only, and they are not designated for *in vitro* diagnostic purposes. However, their apparent usefulness in the clinical consideration of druggable molecular targets within a specific tumor brings forward a demand for their validation also for diagnostic purposes.

## Data Availability Statement

The datasets generated for this study are available on request to the corresponding author.

## Ethics Statement

This study was reviewed and approved by The Research Ethics Committee of the School of Medicine, Masaryk University (Brno, Czech Republic). Written informed consent was obtained from the minor(s)' legal guardian/next of kin for use of the biological samples and corresponding clinical data for research purposes, as well as for the publication of any potentially identifiable images or data included in this article.

## Author Contributions

JN, JSt, and RV designed the study. KP and JSt provided tumor samples and relevant clinical data. JN and PM performed phospho-protein arrays. LK performed immunohistochemical analyses. MK performed statistical analyses. JN and RV composed the manuscript. MK and JSk participated in data analyses and manuscript preparation. All authors reviewed and approved the final version of the manuscript.

### Conflict of Interest

The authors declare that the research was conducted in the absence of any commercial or financial relationships that could be construed as a potential conflict of interest.
