# Supplementary material for: Phospho-Protein Arrays as Effective Tools for Screening Possible Targets for Kinase Inhibitors and Their Use in Precision Pediatric Oncology
Source: Front Oncol. 2019 Sep 20;9:930. doi: 10.3389/fonc.2019.00930 (PMC6763615; doi:10.3389/fonc.2019.00930)
Supplement: Supplementary file 1 [file Data_Sheet_1.pdf]

Supplementary Material

1 Supplementary Data

Proteome Profiler™ Human Phospho-RTK Array Procedure:

- Blocking of the array in 2 ml of Array Buffer 1 for 1 hour at room temperature on a rocking platform shaker
- Incubation of the array with sample overnight at 2-8°C on a rocking platform shaker
- Washing of the array with Wash Buffer for 3 x 10 minutes at room temperature on a rocking platform shaker
- Incubation of the array with Anti-Phospho-Tyrosine-HRP Detection antibody diluted in Array Buffer 2 for 2 hours at room temperature on a rocking platform shaker
- Washing of the array with Wash Buffer for 3 x 10 minutes at room temperature on a rocking platform shaker
- Incubation of the array with Chemi Reagent Mix for 1 minute
- Detection of chemiluminiscence using X-ray film

Detailed working procedure is available on a web of the manufacturer. Link for the download: https://resources.rndsystems.com/pdfs/datasheets/ary001b.pdf

Proteome Profiler™ Human Phospho-MAPK Array Procedure:

- Blocking of the array in 2 ml of Array Buffer 5 for 1 hour at room temperature on a rocking platform shaker
- Incubation of the sample with 20 µl of Detection Antibody Cocktail for 1 hour at room temperature
- Incubation of the array with sample + Detection Antibody Cocktail overnight at 2-8°C on a rocking platform shaker
- Washing of the array with Wash Buffer for 3 x 10 minutes at room temperature on a rocking platform shaker
- Incubation of the array with Streptavidin-HRP diluted in Array Buffer 5 for 30 minutes at room temperature on a rocking platform shaker
- Washing of the array with Wash Buffer for 3 x 10 minutes at room temperature on a rocking platform shaker
- Incubation of the array with Chemi Reagent Mix for 1 minute
- Detection of chemiluminiscence using X-ray film

Detailed working procedure is available on a web of the manufacturer. Link for the download: https://resources.rndsystems.com/pdfs/datasheets/ary002b.pdf

This product is currently discontinued.

2 Supplementary Tables

Supplementary Table 1: List of detected phospho-proteins using the Proteome Profiler™ Human Phospho-RTK Array Kit.

EGFR	ErbB2	ErbB3	ErbB4	FGFR1	FGFR2α	FGFR3
FGFR4	InsR	IGF-IR	Axl	Dtk	Mer	HGFR
MSPR	PDGFRα	PDGFRβ	SCFR	Flt-3	M-CSFR	c-Ret
ROR1	ROR2	Tie-1	Tie-2	TrkA	TrkB	TrkC
VEGFR1	VEGFR2	VEGFR3	MuSK	EphA1	EphA2	EphA3
EphA4	EphA6	EphA7	EphB1	EphB2	EphB4	EphB6
ALK	DDR1	DDR2	EphA5	EphA10	EphB3	RYK

Supplementary Table 2: List of proteins with detected phospho-sites using the Proteome Profiler™ Human Phospho-MAPK Array Kit.

Protein	Phospho-site	Protein	Phospho-site	Protein	Phospho-site
Akt1	S473	HSP27	S78/S82	p38β	T180/Y182
Akt2	S474	JNK1	T183/Y185	p38δ	T180/Y182
Akt3	S472	JNK2	T183/Y185	p38γ	T183/Y185
Akt pan	S473, S474, S472	JNK3	T221/Y223	p53	S46
CREB	S133	JNK pan	T183/Y185, T221/Y223	p70 S6k	T421/S424
ERK1	T202/Y204	MKK3	S218/T222	RSK1	S380
ERK2	T185/Y187	MKK6	S207/T221	RSK2	S386
GSK-3α/β	S21/S9	MSK2	S360	TOR	S2448
GSK-3β	S9	p38α	T180/Y182		

3 Supplementary Figures

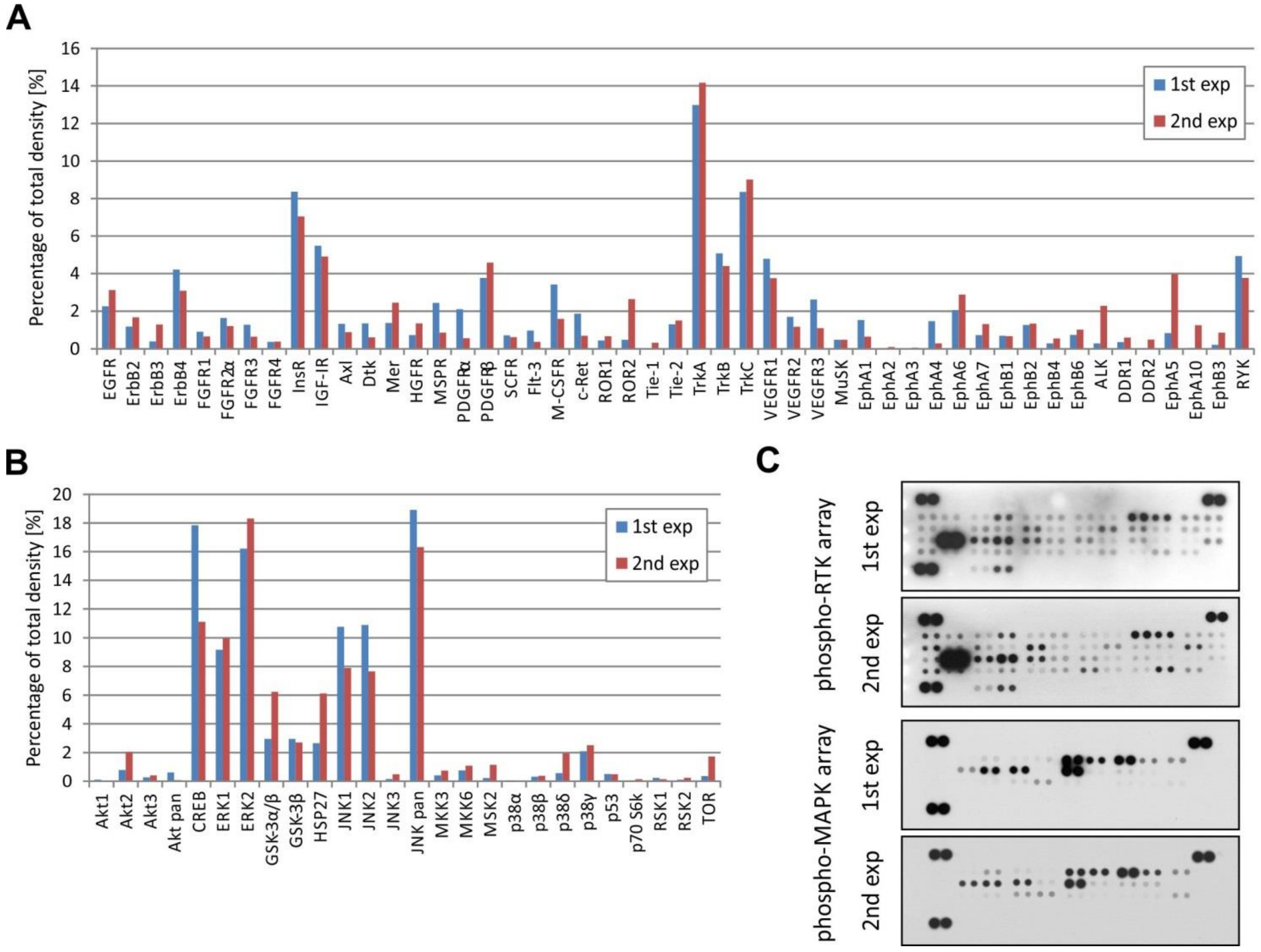

Supplementary Figure 1. Stability of phosphorylation profiles. Repeated analysis of phospho-RTK (A, C) and phospho-MAPK array (B, C) using the tissue sample No. 9 in a time span of 8 months. The array images from the first and second experiment are shown for both arrays (C).
